# Dihydromyricetin Protects against Bone Loss in Ovariectomized Mice by Suppressing Osteoclast Activity

**DOI:** 10.3389/fphar.2017.00928

**Published:** 2017-12-19

**Authors:** Libo Zhao, Cong Cai, Jing Wang, Liming Zhao, Weijin Li, Changyu Liu, Hanfeng Guan, Yuanli Zhu, Jun Xiao

**Affiliations:** ^1^Department of Orthopaedic Surgery, Tongji Hospital, Tongji Medical College, Huazhong University of Science and Technology, Wuhan, China; ^2^Department of Oncology, Renmin Hospital, Wuhan University, Wuhan, China; ^3^Department of Pathology, Tongji Hospital, Tongji Medical College, Huazhong University of Science and Technology, Wuhan, China

**Keywords:** dihydromyricetin, osteoclast, osteoporosis, RANK, NF-κB

## Abstract

Dihydromyricetin (DMY), the main flavonoid component of *Ampelopsis grossedentata*, possesses pharmacological activities useful for treatment of diseases associated with inflammation and oxidative damage. Because osteoclasts are often involved in chronic low-grade systemic inflammation and oxidative damage, we hypothesized that DMY may be an effective treatment for osteoclast-related diseases. The effects of DMY on osteoclast formation and activity were examined *in vitro*. Female C57BL/6 mice were ovariectomized to mimic menopause-induced bone loss and treated with DMY, and femur samples were subjected to bone structure and histological analysis, serum biochemical indicators were also measured. DMY suppressed the activation of nuclear factor-κB, c-Fos and mitogen-activated protein kinase, and prevented production of reactive oxygen species. DMY decreased expression of osteoclast-specific genes, including *Trap, Mmp-9, Cathepsin K, C-Fos, Nfatc1*, and *Rank*. In addition, DMY prevented bone loss and decreased serum levels of tumor necrosis factor-α, interleukin-1β, and interleukin-6, and with a decrease in the ratio between receptor activator of nuclear factor-κB (RANK) ligand (RANKL) and osteoprotegerin (OPG) *in vivo*. These findings demonstrate that DMY attenuates bone loss and inhibits osteoclast formation and activity through modulation of multiple pathways both upstream and downstream of RANKL signaling. DMY may thus be a useful option for treatment of osteoclast-related diseases such as rheumatoid arthritis and osteoporosis.

## Introduction

Osteoporosis is a systemic skeletal disorder characterized by low bone mass and structural deterioration of bone tissue resulting in fragility and susceptibility to fractures, it has become a major public health problem. Bone homeostasis is a delicate balance between bone formation by osteoblasts and bone resorption by osteoclasts (Boyle et al., 2003). Osteoblasts can secrete RANKL and OPG cross talk with osteoclast differentiation, RANKL binds with RANK on preosteoclasts stimulating their differentiation into osteoclasts, and OPG acting as a decoy receptor to bind with RANKL, thus inhibiting the differentiation of osteoclasts (Raggatt and Partridge, 2010). Osteoclasts are multinucleated giant cells formed from the differentiation of a monocyte/macrophage lineage of hematopoietic progenitor cells through a multi-stage process of cell adhesion, proliferation, motility, cell–cell contact, and terminal fusion. Immune cells have been linked to bone loss associated diseases, B-lymphocyte involvement in the adaptive immune response by the upregulation of RANKL expression and T cells of the ability to produce RANKL in the presence of immune stimulus, they control the bone turnover through increasing osteoclastogenesis (Mori et al., 2013, 2015). Macrophage-colony stimulating factor (M-CSF) and RANKL are two key factors for differentiation and function of osteoclasts (Jimi et al., 1999). M-CSF binds to c-Fms/CSF1-R on osteoclast precursor-like cell line and promotes their survival and proliferation through activation of extracellular signal-regulated kinase (ERK) and PI3K/Akt. M-CSF can stimulate RANK expression on osteoclast precursor-like cell line (Kikuta and Ishii, 2013). Chronic inflammatory process can also induce ever increasing osteopenia (Straub et al., 2015). Pro-inflammatory cytokines such as tumor necrosis factor-α (TNF-α), interleukin-6 (IL-6), and interleukin-1β (IL-1β) also stimulate osteoclast differentiation and activity by increasing production of RANKL (Zupan et al., 2012; Boyce et al., 2015). RANKL binds to RANK on osteoclast precursor-like cell line and mature osteoclasts, through TNF receptor-associated factor 6 (TRAF6), leading to the activation of several signaling cascades. The activated signaling pathways include nuclear factor-κB (NF-κB), ERK, c-Jun, N-terminal kinase (JNK), and p38 mitogen-activated protein kinase (MAPK) (Asagiri and Takayanagi, 2007). These pathways play essential roles in osteoclastogenesis, such that influence on any one of them may have profound effects on osteoclast differentiation and bone resorption (Teitelbaum and Ross, 2003).

Flavonoids are members of the catechin family distributed phenolic compounds in plant foods with potential antioxidant agents (Bravo, 1998). Dihydromyricetin (DMY) is the main flavonoid component as the most abundant (approximately 30%) and bioactive constituent of *Ampelopsis grossedentata*, a medicinal and edible plant widely distributed in Southern China (Wang et al., 2002; Jiang et al., 2014). DMY has been reported to possess biological and pharmacological activities, including anti-oxidative, anti-inflammatory, anti-apoptotic, hepatoprotective, and cardioprotective effects (Hou et al., 2015; Li et al., 2015; Chen et al., 2016; Liu et al., 2016). Recent studies have demonstrated that DMY suppresses the levels of pro-inflammatory cytokines such as TNF-α, IL-1β, and IL-6 in lipopolysaccharide-treated mice. DMY exerts its anti-inflammatory action by suppressing the activation of NF-κB and the phosphorylation of p38 and JNK, and it may be a potentially useful therapeutic agent for inflammatory-related diseases (Hou et al., 2015; Tang et al., 2016). Since NF-κB pathways are essential for osteoclastogenesis (Boyle et al., 2003), we propose that there is an impact of DMY in osteoclastogenesis. The present study aimed to investigate the activity of DMY on osteoclast formation and function using both *in vivo* and *in vitro* models.

## Materials and Methods

DMY (HPLC ≥ 98%), the natural flavonoid extracted from *Ampelopsis grossedentata*, was purchased from Sigma–Aldrich and dissolved in DMSO. We chose the dose of DMY according to the literature (Wang et al., 2016). The vehicle (VEH) was added to controls at the same concentration as in the DMY group (maximum: 75 μM).

### Cell Culture and Treatment

RAW264.7, a murine monocytic cell line, was obtained from the Cell Bank of the Chinese Academy of Sciences (Shanghai, China). Bone marrow mononuclear cells (BMMCs) were isolated from the tibial and femoral bone marrow of 6–8 week C57BL/6 mice as we described previously (Guan et al., 2015). Both RAW264.7 and BMMCs were cultured in Dulbecco’s Modified Eagle’s medium (DMEM) supplemented with 10% heat-inactivated fetal bovine serum, streptomycin (100 μg/mL), and penicillin (100 U/mL) at 37°C in a humidified incubator with an atmosphere of 95% air plus 5% CO_2_. The culture medium for BMMCs was supplemented with 25 ng/mL M-CSF (R&D Systems China). For the subsequent experiment, RAW264.7 and BMMCs were treated with RANKL (50 ng/ml, R&D Systems China) and various concentrations of DMY (12.5, 25, 50, or 75 μM) to induce osteoclast formation, and the culture medium was replaced every day.

### TRAP Staining and TRAP Enzyme Activity Assay

Four days after treatment, tartrate resistant acid phosphatase (TRAP) staining was performed on cultured RAW264.7 cells and BMMCs with a TRAP staining kit (Sigma–Aldrich, Shanghai, China) according to the manufacturer’s protocol. TRAP-positive cells with three or more nuclei were counted as osteoclasts (Asagiri and Takayanagi, 2007). Cell images were taken using a digital camera attached to a Nikon ECLIPSE TE2000-S microscope (Nikon, Japan). TRAP enzyme activity was measured with a TRAP Assay Kit (Sigma–Aldrich, Shanghai, China) following the manufacturer’s instructions. Briefly, culture medium was collected from osteoclasts formed by BMMCs. TRAP enzyme activity was measured with a Synergy fluorescence plate reader at 405 nm on a colorimetric plate reader.

### Cell Viability Assay

For cell viability assay, RAW264.7 cells and BMMCs were separately seeded in 96-well plates, the culture medium for BMMCs containing 25 ng/mL M-CSF. After 24 h, cells were treated with DMY as indicated concentrations, the culture medium containing DMY was replaced every day. At the end of the incubation, 100 μl medium with 5 mg/10 ml MTT solution (Sigma–Aldrich, Shanghai, China) were added and incubated at 37°C for an additional 4 h. Then, the medium was removed and 200 μl of DMSO was added to each well. Absorbance was measured at 570 nm with a microplate reader (ChemiDoc MP, Bio-Rad, United States).

### Pit Formation and Actin Ring Formation Assays

Bone marrow mononuclear cells were treated with RANKL (50 ng/ml) and M-CSF (25 ng/mL) on 6-well collagen pre-coated plates for 4 days to form osteoclasts. Then mature osteoclasts were collected using 2.5 mg/mL collagenase in dissociation buffer (Life Technologies, Carlsbad, CA, United States) and seeded onto Corning Osteo Assay Surface (Corning Incorporated Life Science, Corning, NY, United States) in a multiple well plate in the presence of RANKL (50 ng/ml) and different concentrations of DMY supplemented with M-CSF (25 ng/mL) for 3 days. The disks were washed with 5% sodium hypochlorite for 5 min, and images were taken and resorption was quantified by image analysis (Bioquant Image Analysis, Nashville, TN, United States). The actin ring structure formation assay was examined by fluorescence microscopy as described previously (Soysa et al., 2009; Wilson et al., 2009).

### Quantitative RT-PCR

Quantitative real-time polymerase chain reaction (qRT-PCR) was performed as described before (Guan et al., 2010, 2013). Briefly, RAW264.7 cells were treated with RANKL and 75 μM DMY for 3 days, total RNA was isolated using TRIzol reagents (Invitrogen Life Technologies, Carlsbad, CA, United States) and first-strand cDNA was synthesized with MMLV reverse transcriptase (Promega, Madison, WI, United States). Templates were amplified with QuantiTect SYBR Green PCR Kit (Qiagen, Valencia, CA, United States) on the iCycler real-time PCR instrument (BIO-RAD, Berkeley, CA, United States). Primers are listed in **Table 1** (annealing temperature = 60°C).

**Table 1:** Sequences of primers used in the real-time PCR.

Name		Sequences 5′–3′
*Rank*	Forward	CAGGAGAGGCATTATGAGCA
	Reverse	GGTACTTTCCTGGTTCGCAT
*Trap*	Forward	GATGCCAGCGACAAGAGGTT
	Reverse	CATACCAGGGGATGTTGCGAA
*Cathepsin K*	Forward	GAAGAAGACTCACCAGAAGCAG
	Reverse	TCCAGGTTATGGGCAGAGATT
*Mmp-9*	Forward	CTGGACAGCCAGACACTAAAG
	Reverse	CTCGCGGCAAGTCTTCAGAG
*Nfatc1*	Forward	CAACGCCCTGACCACCGATAG
	Reverse	GGGAAGTCAGAAGTGGGTGGA
*C-Fos*	Forward	GGTGAAGACCGTGTCAGGAG
	Reverse	TATTCCGTTCCCTTCGGATT
*β-actin*	Forward	ATTTCTGAATGGCCCAGGT
	Reverse	CTGCCTCAACACCTCAACC

*Rank, receptor activator of NF-κB; *Trap*, tartrate resistant acid phosphatase; *Mmp-9*, matrix metallopeptidase-9; *Nfatc1*, nuclear factor of activated T cells, cytoplasmic 1.*

### Western Blot Analysis and Electrophoretic Mobility Shift Assay

Immunoblots were performed on RAW264.7 cells as described (Guan et al., 2010, 2013). RAW264.7 cells were treated with RANKL and 75 μM DMY for the indicated time, then protein was prepared. The following primary antibodies (1:1000 dilution) were obtained from Cell Signaling (Cell Signaling Technology, Boston, MA, United States): phospho-Akt (Ser-473), Akt, phospho-ERK1/2 (Thr202/Tyr204), ERK, phospho-JNK (Thr183/Tyr185), JNK, phospho-p38 (Thr180/Tyr182), p38. The antibodies (1:500 dilution) against GAPDH and β-Actin were obtained from BOSTER (BOSTER, Wuhan, China). Secondary antibodies (1:5000 dilution) used were goat anti-rabbit IgG-horseradish peroxidase (HRP; sc-2004, Santa Cruz, CA, United States). Signals were visualized with enhanced chemiluminescence and captured by a scanner (ChemiDoc MP, Bio-Rad, United States). Electrophoretic mobility shift assay (EMSA) was performed as described previously using a LightShift Chemiluminescent EMSA Kit (Thermo Fisher Scientific, China) (Guan et al., 2013). Briefly, RAW264.7 cells in 6-well plates were pretreated with DMY with a concentration of 75 μM for 2 h, then stimulated with RANKL (50 ng/ml) for 30 min. Nuclear extracts were prepared using Nuclear and Cytoplasmic Protein Extraction Kit according to the manufacturer’s instructions (Beyotime Institute of Biotechnology, Jiangsu, China) and quantified. An equal amount of nuclear extract was incubated with biotin end-labeled duplex DNA and electrophoresed on a 6% polyacrylamide native gel. The AP-1 and NF-κB probes (Beyotime Institute of Biotechnology, Jiangsu, China) used for EMSA, containing the consensus recognition site were as follows: AP-1, 5′-CGCTTGATGACTCAGCCGGAA-3′; NF-κB, 5′-AGTTGAGGGGACTTTCCCAGGC-3′.

### Measurement of ROS Production

RAW264.7 cells were cultured on 12-well plates, after treatment with DMY for 36 h, cells were incubated for 30 min in presence of RANKL (50 ng/ml). Subsequently, ROS production was measured by flow cytometry with an ROS assay kit (Beyotime Institute of Biotechnology, Jiangsu, China) as described previously (Guan et al., 2015).

### Animals

This study was carried out in accordance with the re-commendations of Animal Experimentation Guidelines, the Ethics Committee on Animal Experimentation of Tongji Medical College, Huazhong University of Science and Technology (Wuhan, China). All animal procedures were approved by the Ethics Committee on Animal Experimentation of Tongji Medical College, Huazhong University of Science and Technology (Wuhan, China). C57/BL6 mice were purchased from the Experimental Animal Center of Tongji Medical College (Wuhan, China). All mice were kept in ventilated filter-top cages under standard laboratory conditions at a constant temperature of 25°C with a 12-h light/dark cycle, and were fed conventional rodent chow with water.

### Ovariectomized Mouse Model

Four-month-old female C57BL/6 mice (21 ± 1 g) were divided randomly into three groups (*n* = 12 mice per group): sham-operated mice, bilateral ovariectomized (OVX) mice treated with VEH, and OVX mice treated with DMY. Sham operation was performed by identifying the bilateral ovaries and ovariectomy was performed by removing the bilateral ovaries, both through a dorsal approach. One day after operation, mice were injected intraperitoneally with VEH or DMY 50 mg/kg/d 5 days a week for 6 weeks. After 6 weeks, the mice were sacrificed for experiments and uterus wet weight was measured to validate the success of ovariectomy.

### Bone Structure Analysis

After removal of soft tissues, microcomputer tomography (μCT) (μ -CT50 ScancoMedical, Bassersdorf, Switzerland) was performed on the distal femur. Scans were taken with a source voltage of 80 kV and 80 μA source current with a voxel size of 10 μm. The bone structural parameters of bone mineral density (BMD), bone volume/tissue volume (BV/TV), trabecular number (Tb.N), trabecular thickness (Tb.Th), and trabecular separation (Tb.Sp) were quantitatively analyzed with the built-in software of the μCT. The three-dimensional bone structure image slices were reconstructed using the built-in software. Nomenclature and abbreviations of parameters follow the recommendations of the American Society of Bone and Mineral Research (Bouxsein et al., 2010).

### Histological Analysis

For histological analysis, the femur samples were decalcified with 10% tetrasodium-EDTA aqueous solution at 4°C for 1 week. The samples were then embedded in paraffin. Paraffin-embedded bone sections (5 μm) of each femur were prepared for hematoxylin and eosin (H&E) staining and TRAP staining to observe the histology of the metaphysis below the primary spongiosa. Histological measurements and images were taken by a microscope equipped with a camera. Trabecular bone density was measured in sections with H&E staining, while numbers of osteoclasts of trabecular bone surface were counted in the sections with TRAP staining.

### Measurement of Serum Biochemical Indicators

Blood from Sham+VEH, OVX+VEH, and OVX+DMY mice was collected by retro-orbital puncture before sacrifice. According to the manufacturer’s instructions, serum levels of TRAP, TNF-α, IL-1β, IL-6, RANKL, and OPG were measured by enzyme-linked immunosorbent assay (ELISA) kits: TRAP ELISA kit (BD Biosciences, San Jose, CA, United States), TNF-α, IL-1β, and IL-6 ELISA kits (eBioscience, San Diego, CA, United States), and RANKL and OPG ELISA kits (Boster, Wuhan, China).

### Statistical Analysis

All experiments were independently repeated three times with similar results. Data are expressed as mean ± standard deviation (SD). Student’s *t*-test was used for comparison between two groups, and analysis of variance (ANOVA) was used in multiple comparisons. All statistical analyses were carried out with SPSS13.0 software (SPSS, Chicago, IL, United States), statistical significance was considered as *P* < 0.05.

## Results

### DMY Inhibits Osteoclast Differentiation, Activity, and Bone Resorption *in Vitro*

**FIGURE 1:**
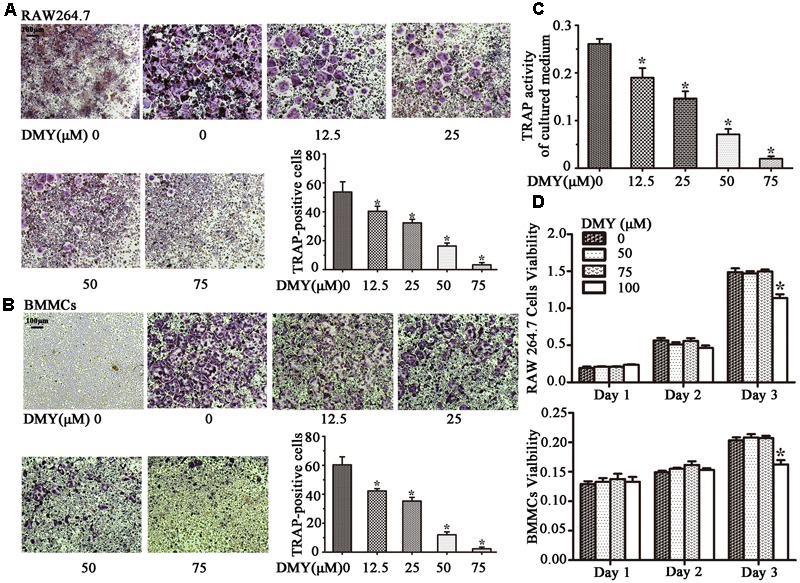
Dihydromyricetin (DMY) inhibits osteoclast differentiation and activity *in vitro*. To induce osteoclast formation, RAW264.7 and BMMCs (supplemented with M-CSF at 25 ng/ml) were treated with RANKL (50 ng/ml) and different concentrations of DMY (12.5, 25, 50, or 75 μM). Four days after treatment, cells were used for TRAP staining and TRAP enzyme activity assay. TRAP positive cells with three or more nuclei were identified as osteoclasts. **(A,B)** DMY inhibits osteoclast formation in a dose-dependent manner. **(C)** TRAP activity in medium from cultured BMMCs was measured. As indicated in the Figures, it is inhibited by DMY in a dose-dependent manner. **(D)** The effects of DMY on RAW264.7 cells and BMMCs viability were determined by a MTT assay. Data are presented as mean ± SD of 3 independent experiments, ^∗^*P* < 0.05 versus DMY (0 μM) group.

**FIGURE 2:**
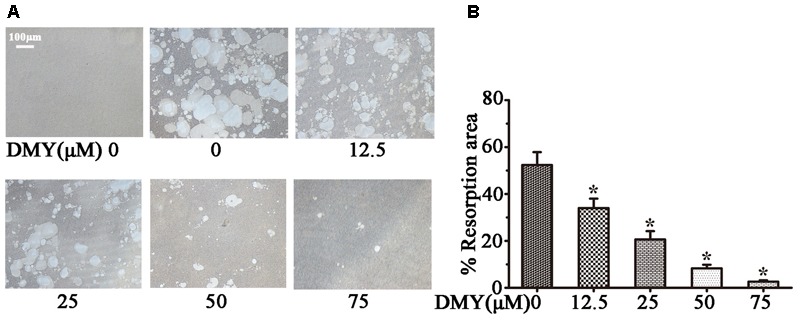
Dihydromyricetin inhibits osteoclast function. DMY inhibited osteoclast bone resorption function. Mature osteoclasts were collected and seeded onto a Corning Osteo Assay Surface. BMMCs were treated with RANKL (50 ng/ml) and M-CSF (25 ng/ml), with or without different concentrations of DMY for 3 days. Images **(A)** were taken and resorption was quantified by image analysis **(B)**. Data are presented as mean ± SD of 3 independent experiments, ^∗^*P* < 0.05 versus DMY (0 μM) group.

**FIGURE 3:**
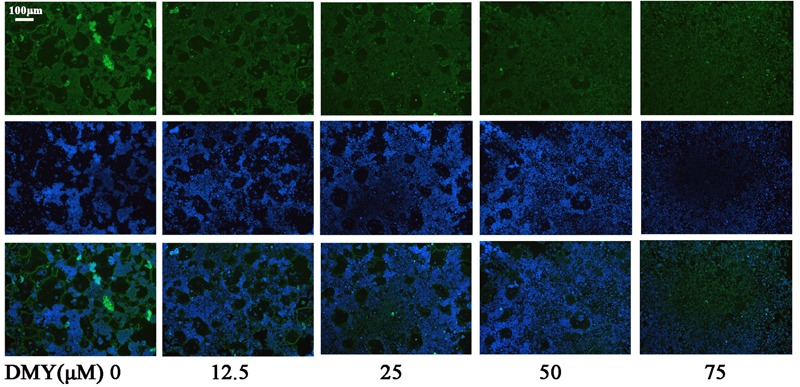
Dihydromyricetin inhibits osteoclast actin ring formation. DMY disrupted the actin ring formation. After culturing BMMCs with RANKL (50 ng/ml), M-CSF (25 ng/ml), and different concentrations of DMY for 4 days, actin ring formation staining was performed and subsequently examined by fluorescence microscopy. Data are of three independent experiments.

Treatment with DMY significantly reduced the numbers of the TRAP-positive multinuclear cells both in RAW264.7 cells (**Figure 1A**) and BMMCs (**Figure 1B**) in a dose-dependent manner, with the maximal effect at 75 μM of concentration. Likewise, DMY treatment decreased TRAP enzyme activity in BMMCs culture medium (**Figure 1C**). The potential toxicity of DMY was evaluated by a MTT assay; DMY up to 75 μM did not detectably inhibit the viability and proliferation of RAW264.7 cells and BMMCs (**Figure 1D**). The effect of DMY on the apoptosis of RAW264.7 cells was also assessed by Annexin V/PI double staining, DMY up to 100 μM did not affect the apoptosis of RAW264.7 cells (Supplementary Figure 1). Pit formation assays were performed to further assess osteoclast function. As shown by the diminished area of resorption pits formed by osteoclasts, osteoclast function was severely impaired by DMY treatment (**Figures 2A,B**). DMY markedly disrupted osteoclast actin ring formation (**Figure 3**), which is essential for the attachment and bone resorption of osteoclasts (Wilson et al., 2009).

### DMY Suppresses Multiple Pathways Involved in Osteoclastogenesis

**FIGURE 4:**
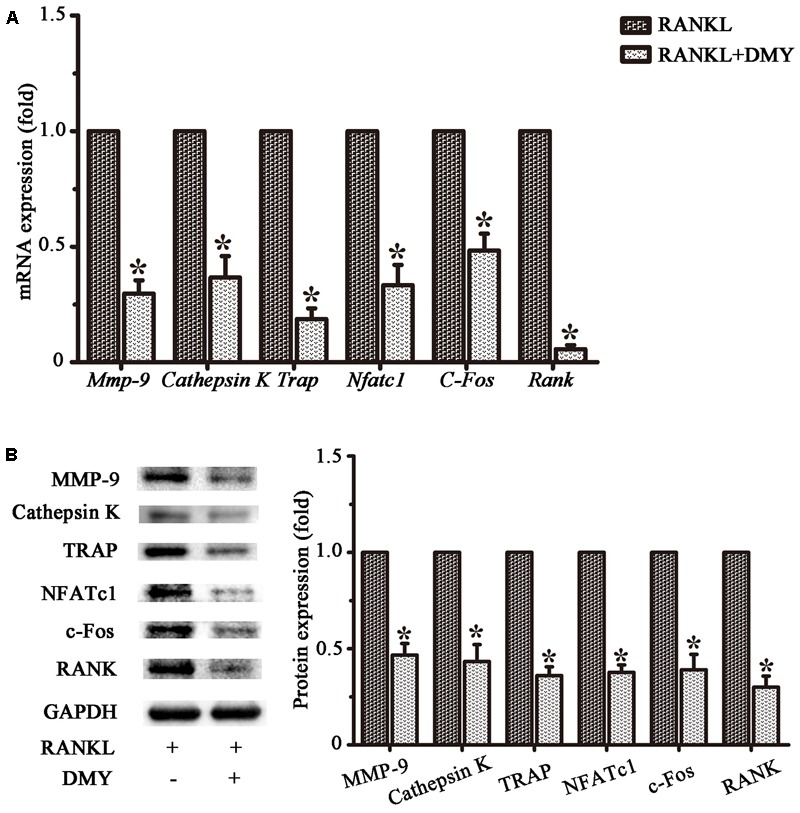
Dihydromyricetin represses expression of osteoclast-specific genes. RAW264.7 cells were treated with RANKL and 75 μM DMY. Cells were collected for total RNA and protein preparation after 3 days. **(A)** Expression of *Mmp-9, Cathepsin K, Rank, Trap, Nfatc1*, and *C-Fos* was determined by qRT-PCR. Values were calculated in relation to the internal control β-actin mRNA by the comparative Ct method. *n* = 3, ^∗^*P* < 0.05. **(B)** Immunoblots with MMP-9, Cathepsin K, RANK, TRAP, NFATc1, and c-Fos antibodies demonstrating that DMY repressed osteoclast-specific markers. The antibody against GAPDH was used as a loading control. Data are presented as mean ± SD of 3 independent experiments, ^∗^*P* < 0.05 versus RANKL group.

**FIGURE 5:**
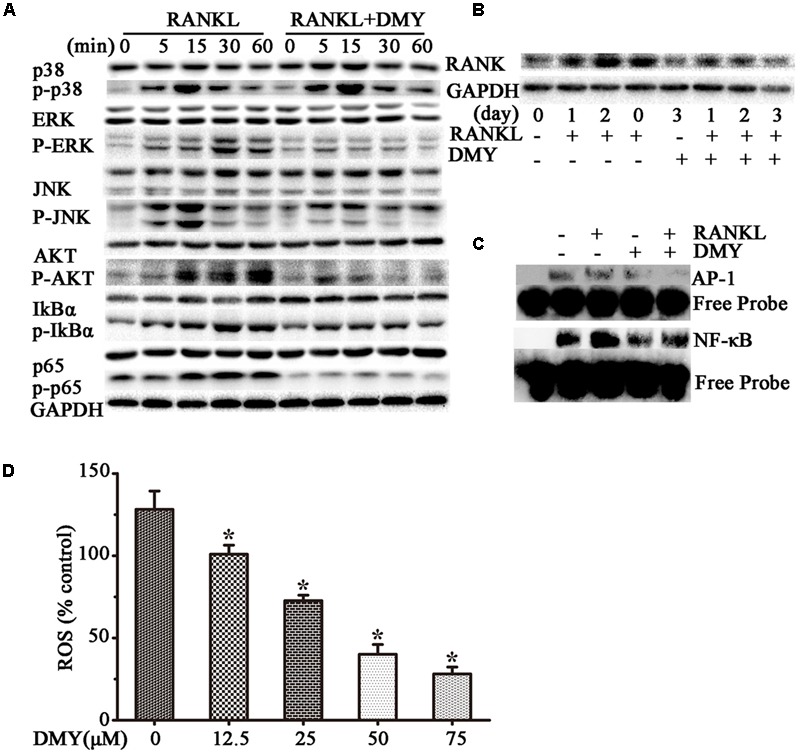
Dihydromyricetin inhibits multiple pathways of osteoclastogenesis. RAW264.7 cells were starved for 12 h before treatment and then pretreated with 75 μM DMY at the indicated concentrations for 2 h. **(A,B)** DMY decreased the expression of multiple osteoclast-specific proteins. Cells were stimulated with RANKL (50 ng/ml) for the indicated time, then protein was extracted for immunoblotting. **(C)** DMY inhibited RANKL-induced NF-κB and AP-1 DNA-binding activity. After pretreatment with DMY for 2 h, RAW264.7 cells were then stimulated with or without 50 ng/m RANKL for 30 min, and nuclear extracts were prepared for EMSA. **(D)** DMY decreased the generation of ROS. By flow cytometry analysis, ROS production was quantified, and DMY decreased the production in dose-dependent manner. The basic ROS level in RAW264.7 cells was set to 1. Data are presented as mean ± SD of 3 independent experiments, ^∗^*P* < 0.05 versus RANKL group.

In RAW264.7 cells, treatment with DMY strikingly inhibited *Trap, Mmp-9, Cathepsin K, Nfatc1, Rank*, and *C-Fos* mRNA expression (**Figure 4A**). Immunoblot analysis demonstrated the downregulation of these osteoclast-specific proteins by DMY (**Figure 4B**). Among the three major subfamilies of MAPK, DMY suppressed RANKL-induced phosphorylation of JNK and ERK (**Figure 5A**). Furthermore, DMY inhibited the levels of AKT phosphorylation (p-AKT), IkBα phosphorylation (p-IkBα), and p65 phosphorylation (p-p65) (**Figure 5A**). The RANK protein level was depressed in our immunoblot analysis (**Figure 5B**).

### DMY Inhibits DNA Binding Activity of NF-κB and AP-1

Transcription factors such as NF-κB and AP-1 play an essential role in osteoclastogenesis (Takayanagi, 2007a,b). By EMSA assays (**Figure 5C**), without RANKL stimulation, DMY had no significant influence on baseline NF-κB or AP-1 DNA-binding activity. Whereas, DMY remarkably inhibited the activation of NF-κB and AP-1 by RANKL.

### DMY Decreases the Release of Intracellular ROS

The intracellular level of ROS is increased by RANKL stimulation, and ROS also activates osteoclast differentiation (Lee et al., 2005). Our results showed that the production of ROS was increased through RANKL stimulation, but was effectively decreased by DMY in a dose-dependent manner (**Figure 5D**).

### DMY Prevents OVX-Induced Bone Loss

**FIGURE 6:**
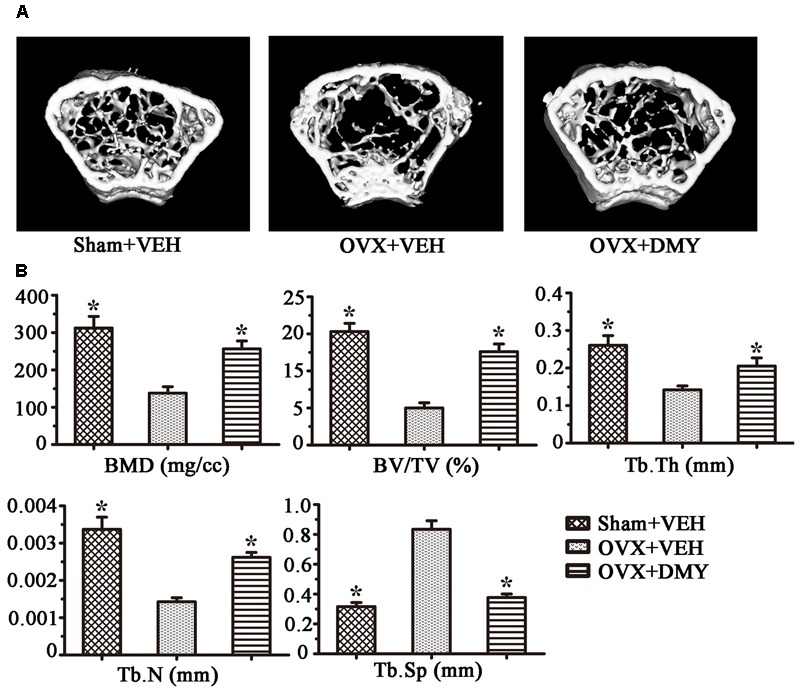
Dihydromyricetin prevents OVX-induced bone loss. OVX mice were sacrificed after 6 weeks of treatment with DMY. μCT images of the distal femur from representative specimens of Sham+VEH, OVX+VEH, and OVX+DMY were obtained, and 3D trabecular architecture was studied using a μCT **(A)**. **(B)** Bone mineral density (BMD), bone value/total value (BV/TV), trabecular number (Tb.N), trabecular thickness (Tb.Th), and trabecular space (Tb.Sp) were analyzed with the built-in software of the μCT. Data are presented as mean ± SD (^∗^*P* < 0.05 versus OVX+VEH group, *n* = 12).

**FIGURE 7:**
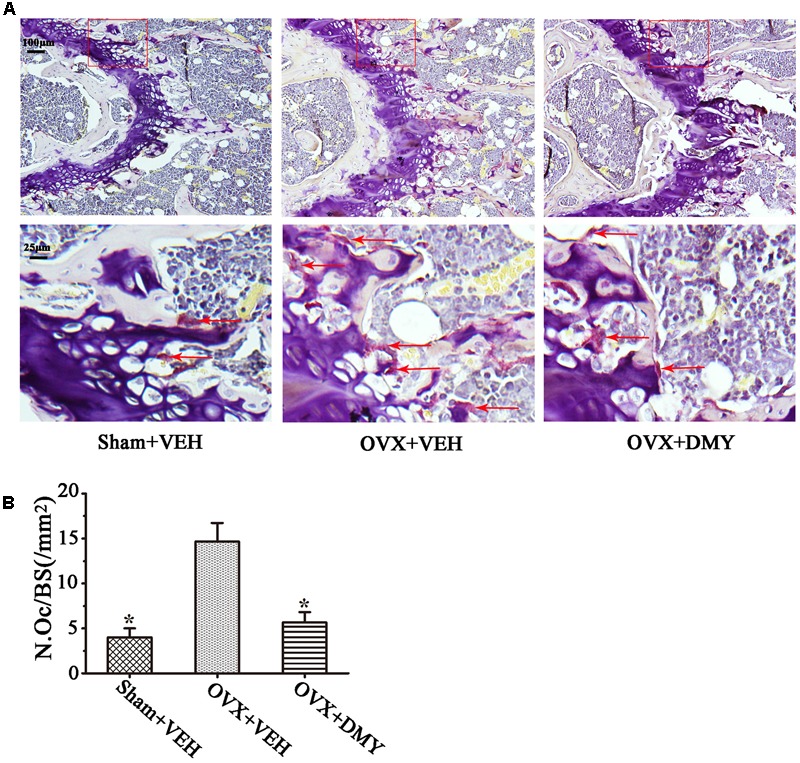
Dihydromyricetin inhibits bone resorption in OVX mice. The paraffin-embedded femoral sections from each group were TRAP stained 6 weeks after operation. **(A)** Sections of the metaphyseal regions of the distal femurs from Sham+VEH, OVX+VEH, and OVX+DMY group were subjected to TRAP staining for visualization of the red-colored TRAP-positive osteoclasts indicated with red arrows in the enlarged images. **(B)** The numbers of osteoclasts (N.Oc) per millimeter of trabecular bone surface (BS) were counted. Data are presented as mean ± SD (^∗^*P* < 0.05 versus OVX+VEH group, *n* = 12).

We evaluated the effects of DMY on osteopenia using an OVX mouse model which mimics menopause-induced bone loss (Bouxsein et al., 2005). The weight of the uterus was used as an indicator of estrogen status to assess the success of ovariectomy and OVX resulted in a significant decrease in uterus weight (Ohlsson et al., 2014) (Supplementary Figure 2). As expected, 6 weeks after operation, treatment with DMY in OVX mice dramatically attenuated trabecular bone loss as shown by the μCT, compared to the sham-operated mice. OVX mice treated with VEH exhibited a significant loss of trabecular bone, as revealed by decreased BMD, BV/TV, Tb.N, and Tb.Th and by increased Tb.Sp (**Figure 6**). The results were further corroborated by H&E (Supplementary Figure 3A) and TRAP staining of decalcified bone sections. Compared with the Sham+VEH group, femoral sections from OVX mice treated with VEH demonstrated a paucity of cancellous bone both proximal and distal to the growth plate. DMY treatment in the OVX mice induced a marked increase in bone density and much fewer TRAP-positive multinucleated cells, which was also shown by the osteoclast numbers per bone surface (N.Oc/BS) (**Figure 7**). Moreover, we further confirmed that DMY (50 mg/kg) did not induce tissue damage *in vivo*, compared with the Sham+VEH group, as no histopathological changes in the liver and kidney tissues were observed in H&E staining after treating with DMY (Supplementary Figures 3B,C).

### DMY Decreases Serologic Markers of Osteoclast Function in OVX Mice

**FIGURE 8:**
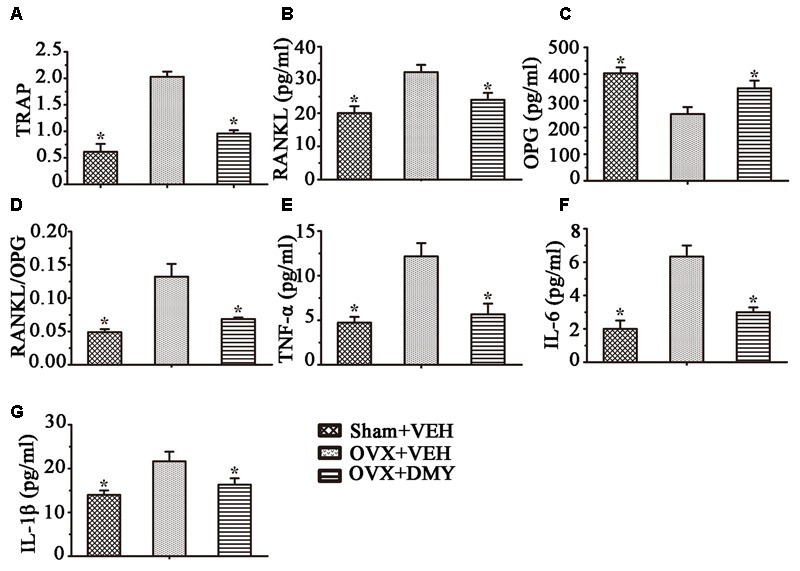
Dihydromyricetin inhibits inflammatory cytokines levels in OVX mice. TRAP activity and levels of RANKL, OPG and inflammatory cytokines in serum were examined using ELISA kits. DMY increased serum OPG, and decreased serum TRAP, RANKL and the RANKL/OPG ratio **(A–D)**. DMY also decreased the levels of TNF-α, IL-1β, and IL-6 in serum **(E–G)**. Data are presented as mean ± SD (^∗^*P* < 0.05 versus OVX+VEH group, *n* = 12).

We next examined the serum levels of TRAP, TNF-α, IL-1β, IL-6, RANKL, and OPG. Serum TRAP level, a serologic marker of osteoclast function, was decreased by DMY (**Figure 8**). These observations suggested that DMY functioned as an inhibitor of osteoclastogenesis and resorption activity. DMY also effectively decreased serum levels of TNF-α, IL-1β, IL-6, and RANKL and increased the serum level of OPG.

## Discussion

**FIGURE 9:**
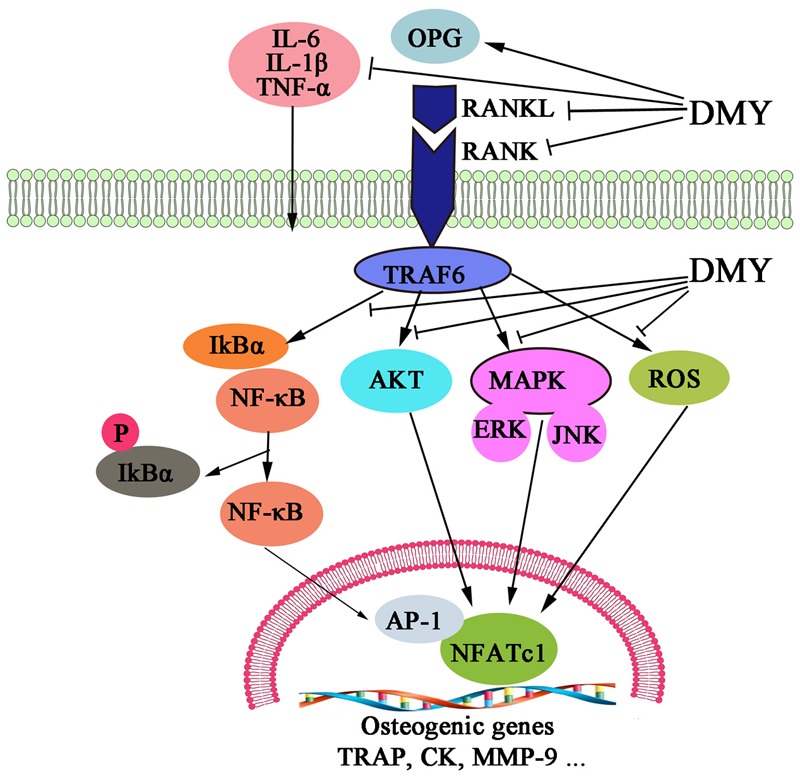
Dihydromyricetin inhibits osteoclastogenesis through multiple pathways. DMY decreased the RANKL:OPG ratio in serum and repressed production of the inflammatory cytokines TNF-α, IL-1β, and IL-6. DMY also repressed multiple pathways downstream of RANKL signaling, including MAPKs, ROS, PI3K/Akt. NF-κB, and AP-1 in osteoclast precursor-like cell line and osteoclasts.

In this study, we showed that DMY efficiently inhibited osteoclastogenesis *in vitro* and ameliorated OVX-induced osteopenia in mice. In our *in vitro* study, DMY inhibited osteoclastogenesis from RAW 264.7 and BMMCs. At the molecular level, DMY profoundly inhibited multiple osteoclast-specific genes including RANK and downstream pathways of RANK signaling, including MAPKs, NF-κB, AP-1, PI3K, and ROS. Moreover, DMY also effectively decreased the serum levels of the inflammatory cytokines TNF-α, IL-1β, and IL-6 and the RANKL/OPG ratio *in vivo*. These data suggest that DMY could inhibit osteoclastogenesis through multiple pathways (**Figure 9**).

Mounting evidence indicates that flavonoids such as DMY have promise in protecting against bone loss (Welch and Hardcastle, 2014; Zhang et al., 2014; Shen and Chyu, 2016). Our results demonstrate that DMY (50 mg/kg/d) could significantly prevent OVX-induced bone loss. However, comprehensive consideration of the pharmacological activities of DMY, the optimal dose for future clinical applications treating osteoporosis maybe deserves further exploration. One study shows that DMY can enhance human bone marrow mesenchymal stem cells osteogenic differentiation *in vitro* partly through Wnt/β-catenin pathway (Zhang et al., 2016). But the role DMY plays in osteoclastogenesis has not been revealed. The classical NF-κB pathway plays an important role in osteoclast formation, differentiation, and bone-resorbing activity, and RANKL-RANK signaling is indispensable for osteoclastogenesis by activation of downstream pathways, including NF-κB and MAPK (Soysa et al., 2009). Studies also show that DMY exerts its anti-inflammatory action through suppressing the activation of NF-κB and MAPK signaling pathways (Hou et al., 2015; Tang et al., 2016). This hints that NF-κB may be the pathway by which DMY suppresses osteoclastogenesis, as our results suggest DMY inhibits multiple downstream pathways of RANK signaling including NF-κB and the phosphorylation of ERK and JNK. Previous studies found that DMY activates PI3K/AKT signaling demonstrated by increased AKT phosphorylation (Liu et al., 2016); PI3K/Akt pathways are also required for osteoclast formation (Tsubaki et al., 2014). Nevertheless, our results show that DMY had inhibited the PI3K/AKT pathway, which is in contrast to previous results. We suppose this might be due to interaction with the RANKL-RANK signaling.

We found DMY also directly inhibited bone-resorbing activity of mature osteoclasts as evidenced by the disruption of osteoclast actin ring structure formation. The underlying mechanism might be the inhibition of DMY on TRAP, MMP-9, and Cathepsin K expression, which are matrix-degrading enzymes essential for degradation of bone (Edwards and Mundy, 2011).

Moreover, ROS acts as a second messenger in cell signaling and the generation of intracellular ROS is increased when RANKL binds to its receptor RANK on the cell surface of osteoclast precursor-like cell line (Shi et al., 2015; Shen and Chyu, 2016). Low levels of ROS may stimulate osteoclast differentiation and bone resorption (Sharma et al., 2014; Shi et al., 2015). DMY has been reported to possess anti-oxidative properties and can effectively decrease intracellular ROS in melanoma cells (Huang et al., 2016; Zhou et al., 2017). However, its effect on osteoclast precursor-like cell line is unclear. We investigated its anti-oxidative activity in osteoclast precursor-like cell line by monitoring ROS production and found that DMY can scavenge ROS, which is consistent with other studies. Furthermore, IL-1 and TNF-α could induce the expression of cyclooxygenase-2 (COX-2), which leading to accelerated osteoclastogenesis as a result of interaction of RANKL with RANK on osteoclast progenitors (Kanematsu et al., 2000). DMY plays anti-inflammatory effects via suppression of COX-2 protein expression (Hou et al., 2015; Wang et al., 2016). Our *in vivo* results showed that DMY decreased the levels of the pro-inflammatory cytokines TNF-α, IL-1β, and IL-6 and the RANK:OPG ratio in serum, which might indirectly attenuate osteoclastogenesis, since these inflammatory cytokines promote osteoclastogenesis and bone resorption (Yu et al., 2004; Mundy, 2007). Considering no significant effect on the uterus weight in OVX mice treated with DMY, it may also deserve further study as one type of estrogen replacement therapy with fewer side effects.

Several limitations of our current study should be noted. The imbalance of bone formation and resorption can both contribute to osteopenia. Though DMY enhances the osteogenic differentiation of human bone marrow mesenchymal stem cells (Zhang et al., 2016), we concentrated only on osteoclasts. Unfortunately, we could not determine its effect on bone marrow mesenchymal stem cells in mice, and further study is needed to validate whether DMY is necessary for osteoblastogenesis from mesenchymal stem cells. Second, while our *in vivo* study showed DMY can prevent OVX-induced bone loss, we did not explore the effects on osteoblastogenesis and bone formation, which also contributed to bone homeostasis. In addition, we used only RAW 264.7 cells for the molecular studies on top of the PI3K pathway, the signaling mechanism regulating activation or inhibition of the PI3K pathway by DMY in different cell types needs to be further clarified.

## Conclusion

Our findings demonstrate that DMY may inhibit osteoclastogenesis through direct and indirect effects to abrogate osteoclast formation and prevent bone destruction. Considering that DMY has been reported to possess numerous biological and pharmacological activities, whether the results could be translated into clinical benefits for patients deserves further study.

## Author Contributions

Study design: LZ and JX. Study conduct: LZ, CC, JW, LmZ, WL, CL, YZ, HG, and JX. Data collection: CC and JW. Data analysis: LZ, YZ, and JX. Data interpretation: YZ and JX. Drafted the manuscript: LZ, YZ, and JX. Approved the final version of manuscript: all the authors. YZ and JX take responsibility for the integrity of the data analysis.

## Conflict of Interest Statement

The authors declare that the research was conducted in the absence of any commercial or financial relationships that could be construed as a potential conflict of interest.
