# Supplementary material for: Dihydromyricetin Protects against Bone Loss in Ovariectomized Mice by Suppressing Osteoclast Activity
Source: Front Pharmacol. 2017 Dec 19;8:928. doi: 10.3389/fphar.2017.00928 (PMC5742133; doi:10.3389/fphar.2017.00928)
Supplement: Supplementary file 1 [file Presentation_1.pdf]

Supplemental Figure Legends

Supplemental Figure 1

Effect of DMY on the apoptosis of RAW264.7 cells. RAW264.7 cells were pretreated with DMY (0 μM, 50 μM, 75 μM or 100 μM) for indicated time. Annexin V-FITC and PI were respectively diluted with 1 × Binding Buffer to the concentrations of 0.5 μg/ml and 0.6 μg/ml. Subsequently, the cells were washed with pre-cold PBS for 3 times, stained in 0.5 μg/ml Annexin V-FITC solution for 15 min and in 0.6 μg/ml PI solution for 5 min, then rinsed with 1 × Binding Buffer for 3 times, followed by analysis using a flow cytometer. The early apoptotic cell (Annexin V^{+} and PI^{-}) were displayed in the lower right quadrant and late apoptotic cells (Annexin V^{+} and PI^{+}) were shown in the upper right quadrant. Data are presented as mean ±SD of 3 independent experiments, $*P > 0.05$ versus DMY (0 μM) group.

Supplemental Figure 2

Six weeks after OVX or sham operation, mice were sacrificed and the uterus wet weight was measured to validate the success of ovariectomy. The % of weight in the Sham + VEH group was set to 1. n = 12, $*P < 0.05$.

Supplemental Figure 3

Six weeks after operation, the paraffin-embedded femoral sections from each group were H&E stained (A). Liver (B) and kidney (C) tissues were fixed in formalin, embedded in paraffin and sectioned at 4 μm thickness. The sectioned samples were then deparaffinized, rehydrated. Liver and kidney sections from sham-operated and all OVX mice were stained with H&E, and observed under a microscope.
