# Supplementary figures and images for: Dihydromyricetin Protects against Bone Loss in Ovariectomized Mice by Suppressing Osteoclast Activity
Source: Front Pharmacol. 2017 Dec 19;8:928. doi: 10.3389/fphar.2017.00928 (PMC5742133; doi:10.3389/fphar.2017.00928)

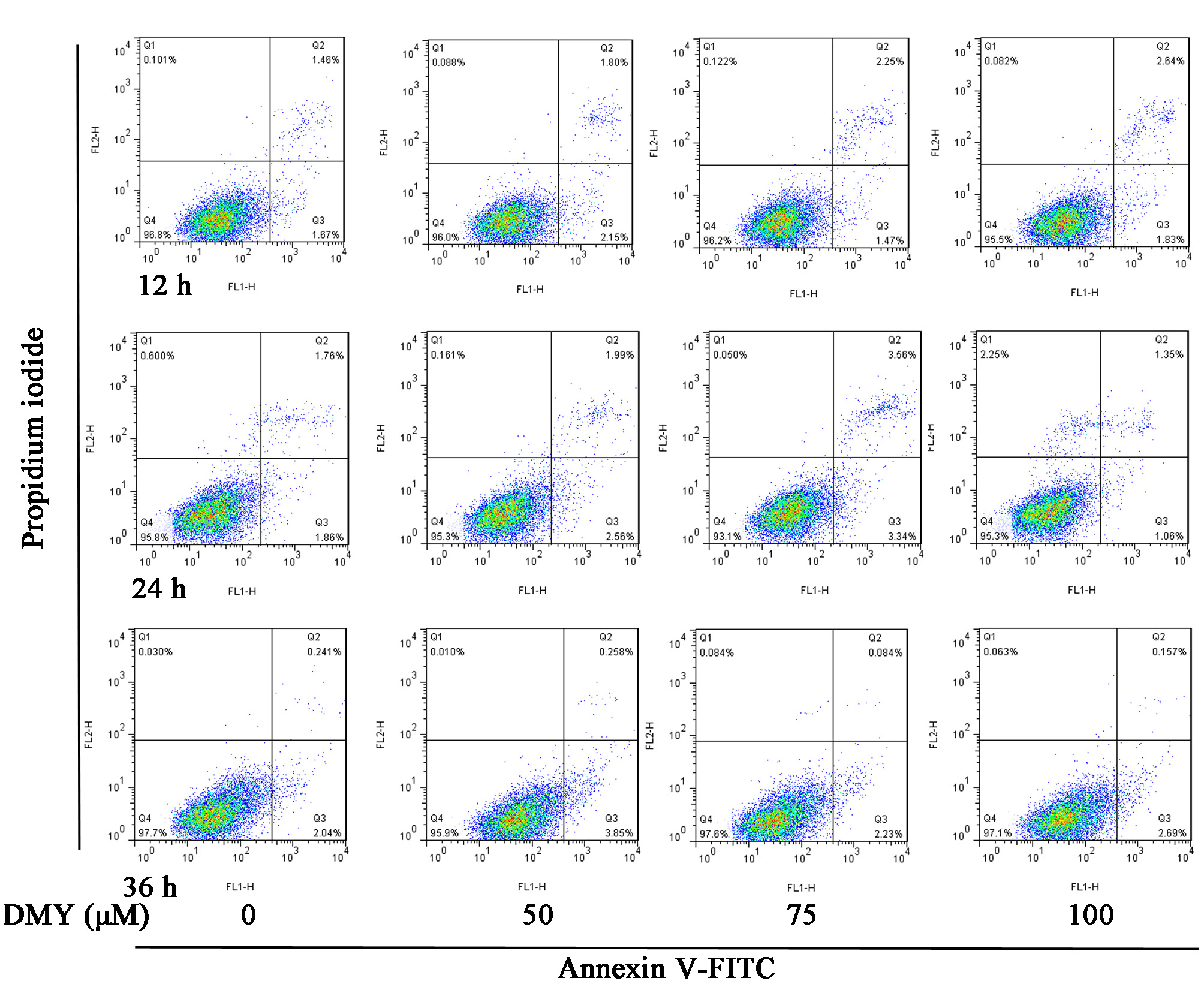

Supplement: Supplementary file 2 [file Image_1.JPEG]

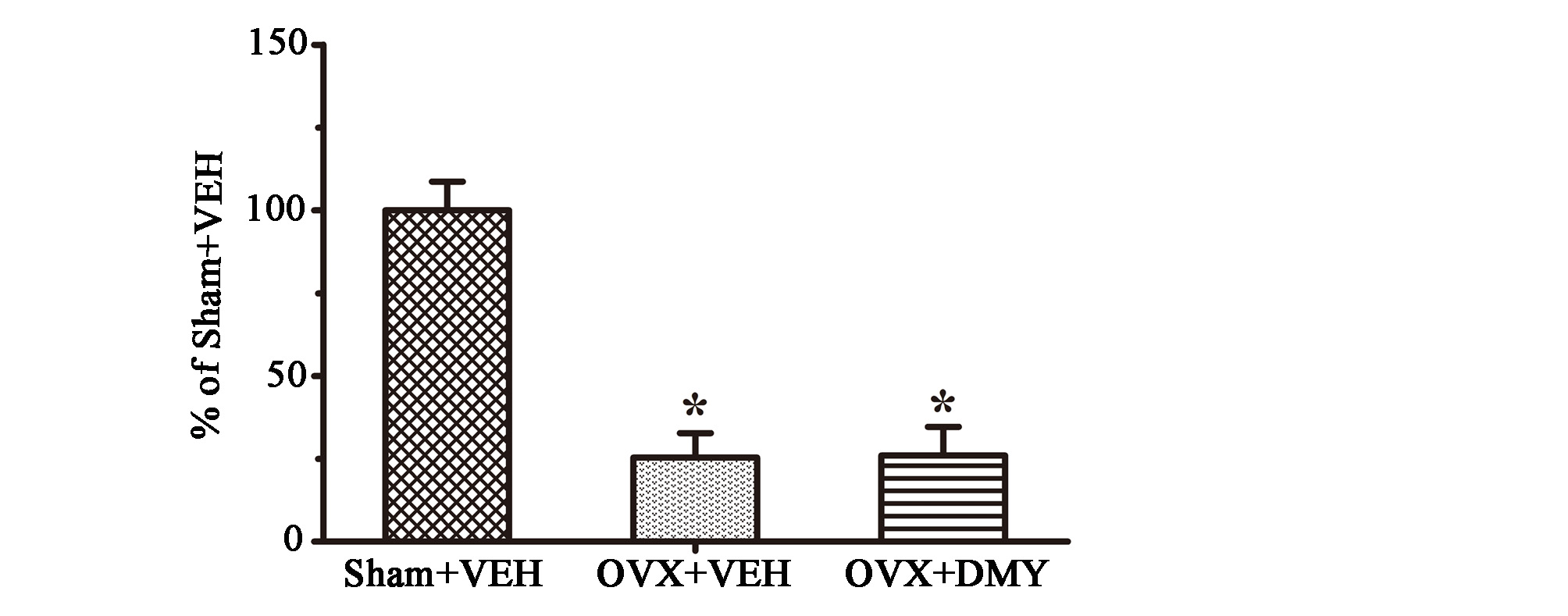

Supplement: Supplementary file 3 [file Image_2.JPEG]

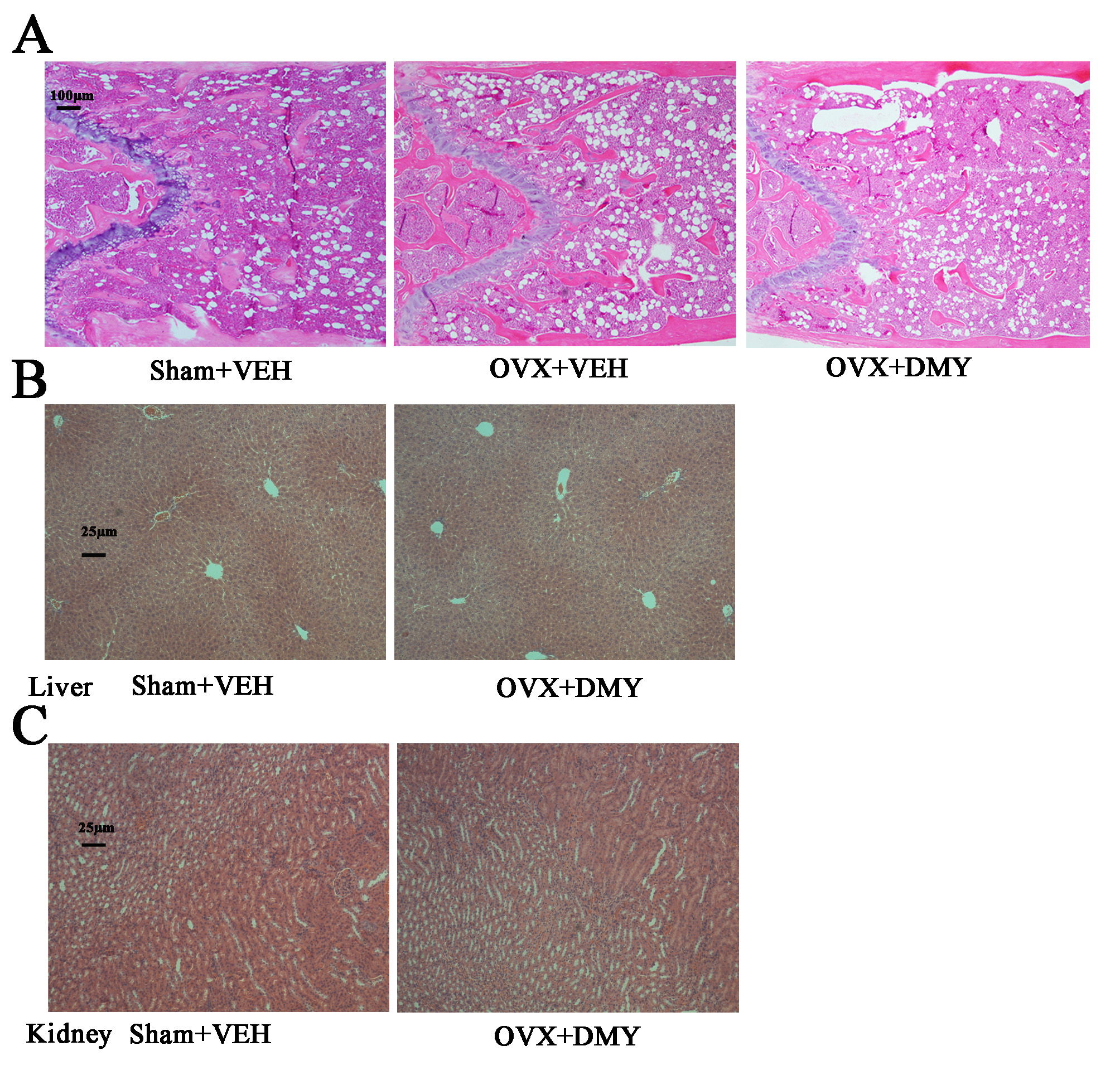

Supplement: Supplementary file 4 [file Image_3.JPEG]
